# Binaphthyl Mediated Low Temperature Synthesis of Carbon Nitride Photocatalyst for Photocatalytic Hydrogen Evolution

**DOI:** 10.1002/cssc.202400618

**Published:** 2024-07-31

**Authors:** Simona Baluchová, Sonia Zoltowska, Paolo Giusto, Baris Kumru

**Affiliations:** ^1^ Department of Analytical Chemistry Faculty of Science Charles University Albertov 6 Prague 2 CZ 128 00 Czech Republic; ^2^ Department of Colloid Chemistry Max Planck Institute of Colloids and Interfaces Am Mühlenberg 1 14476 Potsdam Germany; ^3^ Aerospace Structures & Materials Department Faculty of Aerospace Engineering Delft University of Technology 2629 HS Delft The Netherlands

**Keywords:** carbon nitride, metal-free photocatalysis, organomodified carbon nitride, low temperature carbon nitride, sustainable photocatalyst

## Abstract

Metal‐free graphitic carbon nitrides are on the rise as polymer photocatalysts under visible light illumination, taking shares in a range of promising photocatalytic reactions, including water splitting. Their simple synthesis and facile structural modification afford them exceptional tunability, enabling the creation of photocatalysts with distinct properties. While their metal‐free nature marks a significant step towards environmental sustainability, the high energy consumption required to produce carbon nitride photocatalysts remains a substantial barrier to their widespread adoption. Furthermore, the process of condensation at approximately 550 °C typically results in solid yields of less than 15 %, significantly challenging their economic viability. Here, we report on lowering manufacturing conditions of carbon nitride photocatalysts whilst enhancing photocatalytic activity by introducing binaphthyl diamine as a structural mediator. At 450 °C in 2 hours, carbon nitride photocatalyst shows a lower bandgap and enables visible light induced hydrogen evolution (194 μmol h^−1^) comparable to benchmark carbon nitride photocatalysts.

## Graphical abstract

Research in metal‐free hydrogen generation using graphitic carbon nitride under visible light is promising but faces high temperature ‐low yield syntheses. This study introduces a method to synthesize photocatalytic carbon nitrides at 450 °C using binaphthyldiamine as a structural modifier, which enhances photocatalytic activity and solid yield by overcoming high energy synthesis requirement.

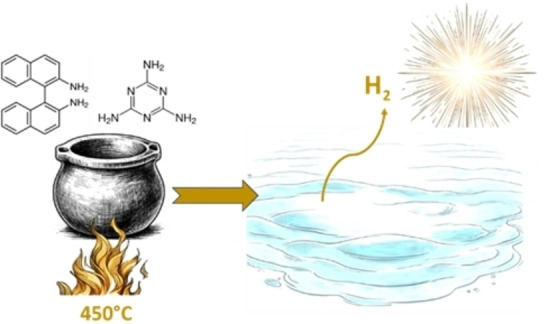

## Introduction

Photocatalytic hydrogen evolution represents a promising pathway to convert solar energy into clean, renewable hydrogen fuel.^[1]^ At the core of this process is the use of photocatalysts–materials capable of absorbing sunlight and using that energy to drive the splitting of water molecules into hydrogen and oxygen.^[2]^ This technique capitalizes on the solar spectrum irradiance, diminishing reliance on fossil fuels and reducing carbon emissions.^[3]^ For effective sunlight absorption and water splitting, the band gap of the semiconductor must align with the solar spectrum, typically ranging from 1.5 to 3.0 electron volts (eV). Additionally, the semiconductor must possess suitable conduction and valence band positions that facilitate at least one of hydrogen or oxygen evolution reactions. Titanium dioxide (TiO_2_) has historically been among the most studied semiconductor for photocatalytic water splitting due to its stability and extensive availability.^[4]^ However, its wide band gap (3.2 eV for anatase phase) limits its absorption to the UV region. This limitation has spurred research into other semiconductor materials, such as cadmium sulfide (CdS),^[5]^ perovskites^[6]^ and zinc oxide (ZnO),^[7]^ yet the presence of metals in these alternatives raises concerns over their environmental impact and hampers their broader adoption for sustainable use.^[8]^

The quest for sustainable photocatalysis has illuminated the potential of graphitic carbon nitrides (g‐CN, C_3_N_4_) as a promising candidate in the field.^[9]^ Characterized by their metal‐free composition and remarkable adaptability, carbon nitrides embody a vast spectrum of polymer photocatalysts.^[10]^ Their ability to absorb visible light and their structure, composed of aromatic tri‐s‐triazine or heptazine units,^[11]^ make them versatile agents for critical applications such as photocatalytic water splitting,^[12]^ CO_2_ photoreduction,^[13]^ ionotronics,^[14]^ interface stabilization^[15]^ and photoredox‐assisted organic^[16]^ and polymer synthesis.[^17^, ^18^] The synthesis of g‐CN is founded on the thermal condensation of nitrogen‐rich organic molecules, like melamine, at temperatures surpassing 500 °C, leading to the formation of the polymer unit and the active photocatalyst.^[19]^ Conversely, at temperatures below 500 °C, the material remains in an oligomer state, characterized by its high luminescence yet lacking photoactivity.^[20]^ Innovations in the field have led to a plethora of tailored structural modifications including enhanced porosity, heteroatom insertion, edge modification, surface charge adjustment, crystallinity enhancement, and variations in the repeating unit, all of which have shown remarkable success in optimizing photocatalytic performance.^[21]^ Molten state carbon synthesis generates organized layers as molten state enables a solvent‐like media for better organization of oligomers during condensation hence improving photocatalytic properties due to enriched interfacial charge transfer processes.^[22]^ Mainly, inorganic deep eutectics such as NaCl/KCl^[23]^ and KNO_3_/KCl^[24]^ and inorganic salt such as silicotungstic acid^[25]^ are employed as structural mediators, yet introducing inorganic atoms into carbon nitride structure. Supramolecular assembly of organic co‐monomers, one of those innovations, offers an organized monomer unit to be condensed around 550 °C for tailored carbon nitride architectures.^[26]^ Typically, two or more nitrogen‐containing monomers can form self‐assembled structures through hydrogen bonding,^[27]^ which allows core modification,^[28]^ as well as edge modification.^[29]^

**Scheme 1:**
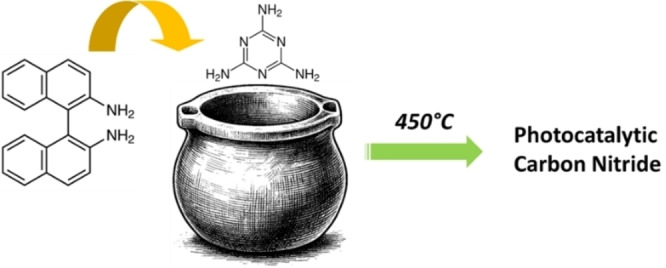
Chemical structures of molecules and conditions employed to fabricate photocatalytic carbon nitride in this study.

The innovative strategy of hydrogen‐bonded co‐monomer assembly unveils remarkable opportunities to customize the properties of carbon nitride. In this study, we highlight the role of binaphthyl diamine as a pivotal structure mediator. This approach significantly streamlines the synthetic process with enhanced solid yield, enabling the production of a photoactive polymer semiconductor under milder conditions (Scheme 1).

## Experimental Details

### Materials

All chemicals were used as purchased. 1,1′‐Binaphthyl‐2,2′‐diamine (97 %, TCI), 1H‐Benzo[g] indole (97 %, Sigma Aldrich), chloroplatinic acid hexahydrate (H_2_PtCl_6_, synthesis grade, Sigma Aldrich), melamine (99 %, Alfa Aesar), poly(dimethylsiloxane), bis(3‐aminopropyl) terminated (*M_n_* 2500, Sigma Aldrich), triethanolamine (TEA, puriss 99 %, Sigma Aldrich).

### Synthesis of Bina‐CN

Melamine (3 g) is mixed with 1,1′‐Binaphthyl‐2,2′‐diamine (1 g) in distilled water and dispersion is mixed overnight to form hydrogen bonded monomer assembly. Afterwards, water is evaporated in a drying oven at 80 °C until a white solid is obtained. Solid is placed in a capped aluminum crucible and heated up to 450 °C (2 h to reach 450 °C and kept 2 h at 450 °C) under a nitrogen‐protected oven. After cooling down, the catalyst is washed with water overnight, filtered, and dried (the catalyst is denoted as Bina‐CN). Solid yield: 44 %.

### Synthesis of Reference g‐CN

10 g of melamine, utilized as a precursor, is placed in a capped aluminum crucible and heated up to 550 °C (4 h to reach 550 °C and kept 4 h at 550 °C) under a nitrogen‐protected oven. After cooling down, the catalyst is washed with water overnight, filtered, and dried (the catalyst is denoted as g‐CN). Solid yield: 12 %.

### Synthesis of Reference 3Bina‐CN

Melamine (3 g) is mixed with 1,1′‐Binaphthyl‐2,2′‐diamine (3 g) in distilled water and dispersion is mixed overnight to form hydrogen bonded monomer assembly. Afterwards, water is evaporated in a drying oven at 80 °C until a white solid is obtained. Solid is placed in a capped aluminum crucible and heated up to 450 °C (2 h to reach 450 °C and kept 2 h at 450 °C) under a nitrogen‐protected oven. After cooling down, the solid is washed with water overnight, filtered, and dried for TGA analysis (the solid is denoted as 3Bina‐CN).

### Synthesis of Reference BI‐CN

Melamine (3 g) is mixed with 1H‐Benzo[g] indole (1 g) in distilled water and dispersion is mixed overnight. Afterwards, water is evaporated in a drying oven at 80 °C until a white solid is obtained. Solid is placed in a capped aluminum crucible and heated up to 450 °C (2 h to reach 450 °C and kept 2 h at 450 °C) under a nitrogen‐protected oven. After cooling down, the solid is washed with water overnight, filtered, and dried (the solid is denoted as Bina‐CN).

### Synthesis of Reference PDMS‐CN

Melamine (3 g) is mixed with bis(3‐aminopropyl) terminated poly(dimethylsiloxane) (1 g) and dispersion is mixed overnight. Dispersion is placed in a capped aluminum crucible and heated up to 450 °C (2 h to reach 450 °C and kept 2 h at 450 °C) under a nitrogen‐protected oven. After cooling down, the solid is washed with water overnight, filtered, and dried (the solid is denoted as PDMS‐CN).

### Photocatalytic Water Splitting Reaction

200 mg of Bina‐CN and 0.5 % Pt salt or 200 mg g‐CN with 1.5 % Pt salt were added in 10 vol.% TEA in water (100 mL). Then, the mixture was illuminated by a 100 W visible LED lamp for 30 minutes. This resulted in photodeposition of Pt nanoparticle on CN powders as commonly employed in literature. Photocatalytic H_2_ production was carried out in a Pyrex top‐irradiation reaction vessel connected to a glass closed gas circulation system. The wavelength of the incident light was controlled by using an appropriate long pass cut‐off filter and applied wavelength was 420 nm. The temperature of the reactant solution was maintained at room temperature by a flow of cooling water during the reaction. The evolved gases from the photocatalytic reaction were analysed using a Chrompack CP 9001 gas chromatograph (GC) equipped with a thermal conductive detector. The setup employs a 500 W Mercury‐ Xenon light source by Newport (Model 66983–500HX−R15). For this, argon was used as carrier gas. The production rates of hydrogen were obtained by converting the area in the obtained chromatograms using a known calibration factor for this specific setup and reactor size. This calibration was obtained by having a known amount of gas in the reactor, after which this gas would be diluted by the carrier gas. Stability tests were performed by venting gasses from reaction media at certain time intervals and subsequently continuing the photocatalytic reaction. Reusability test for 0.5 wt.% Pt loaded Bina‐CN was conducted for 4 independent cycles, where each cycle contains a photocatalytic reaction for 4 hours, recovering the catalyst via centrifugation, washing with water, drying overnight and starting a new cycle. H_2_ evolution apparent quantum yield (AQY) was calculated as follows:

$$AQY\;\left(\%\right)=\left[\left(2\times rH2\times NA\times h\times c\right)/\left(S\times I\times \lambda \right)\right]\times 100$$

where, r_H2_ is the production rate of H_2_ molecules (mol s^−1^), N_A_ is Avogadro constant (6.022×10^23^ mol^−1^), h is the Planck constant (6.626×10^−34^ J s^−1^), c is the speed of light (3×10^8^ m s^−1^), S is the irradiation area (cm^2^), I is the intensity of irradiation light (W cm^−2^) and λ is the wavelength of the monochromatic light (nm).

### Characterization

Powder XRD patterns were obtained using a Bruker D8 ADVANCE X‐ray diffractometer via Cu Kα radiation. SEM were performed using JEOL JSM‐ 7500F equipped with an Oxford Instruments X–MAX 80 mm^2^ detector for the determination of the morphology. Solid‐state ultraviolet−visible (UV−Vis) spectroscopy for powder catalysts was recorded via a Cary 500 scan spectrophotometer equipped with an integrating sphere. FT‐IR spectra were taken on a Nicolet iS 5 FT‐IR spectrometer. Physisorption measurements were performed on a Quantachrome Quadrasorb SI (Austria) at 77 K using N_2_. Samples were degassed overnight before the measurements. Combustive elemental analysis of Bina‐CN and g‐CN was performed via a Vario Micro device. PL emission spectra were recorded on a Jasco FP‐8300 instrument at ambient temperature using solid integrating sphere loaded with catalysts with the excitation wavelength at 360 nm. Emission lifetime decays were determined using C‐SPC on a PicoQuant FluoTime 250 system, with excitation provided by a 375 nm laser diode. Samples were evaluated at the wavelength corresponding to its maximum light emission, as identified by photoluminescence (PL) experiments. The average amplitude lifetimes were determined through a stretched exponential fitting, a method typically used for carbon nitride materials,[^16^, ^30^] using the equation $\bar{\tau }=\frac{\sum a_{i}\tau_{i}}{{\sum a}_{i}}$ . HR‐TEM and STEM measurements were carried out using a double‐Cs‐corrected JEOL ARM200F, equipped with a cold field emission gun and a Gatan GIF Quantum and operated with an acceleration voltage of 200 kV. Samples for HRTEM and STEM were drop‐casted on grids from ethanol dispersions. FFT profiling was generated on Gatan software. TGA was performed via TG 209 Libra from NETZSCH in a nitrogen atmosphere with a heating rate of 10 °C min^−1^ using an aluminum crucible for samples, whereas monomer complex was measured with 1 °C min^−1^. Contact angle measurements were performed on CN deposited on glass surfaces. Bina‐CN and g‐CN (0.5 wt.%) were dispersed in ethanol (5 mL) and sonicated in sonic bath for 3 hours to obtain a colloidal dispersion. Dispersion is coated on glass surface and ethanol is removed at room temperature overnight in fume hood. Sessile drop contact angles of water on the coatings were measured at room temperature about 10 s after placing the drop on the surface with a DSA 10 video contact angle measuring system G10 (Krüss, Germany), and data evaluation was done with DSA version 1.80.02 software. EPR measurements were conducted on a Bruker EMXnano benchtop X‐Band EPR spectrometer. Center Field 3444.05 G, Sweep Width 200 G, Receiver Gain 60 dB, Modulation Amplitude 1.000 G, Number of Scans 4, Microwave Attenuation 10 dB. Sample was placed and flame‐sealed in EPR capillaries (IntraMark), inside EPR tubes (ID 3 mm, OD 4 mm, length 250 mm). EPR measurements of photocatalytic experiments were performed by coupling Thorlabs M415F3 Fiber‐Coupled LED with Thorlabs DC2200 High‐Power LED controller.

Prior to modification procedure, a glassy carbon electrode (GCE; 2 mm diameter; Metrohm, Czech Republic) polished to a mirror finish using an alumina slurry and then washed several times with deionized water. Bina‐CN and g‐CN powders (5 mg) were dispersed in deionized water (5 mL) through stirring and ultrasonication. Subsequently, 6 μL of such suspension was drop‐casted onto the GCE surface and allowed to dry at room temperature for 2 hours. Electrochemical impedance spectroscopy (EIS) measurements were performed at laboratory temperature (23±1 °C) using a μAutolab type III potentiostat equipped with an FRA module and controlled by Nova 2.1.5 software (Metrohm, Czech Republic). The GCE, modified with either Bina‐CN or g‐CN, served as the working electrode in a conventional three‐electrode set‐up, complemented by a silver‐silver chloride reference electrode (Ag|AgCl|3 mol L^−1^ KCl)) and a platinum wire counter electrode (both supplied by Elektrochemické detektory, Czech Republic). EIS was conducted at open circuit potential (OCP) in a solution containing 1 mmol L^−1^ [Fe(CN)_6_] ^3−^ and 1 mmol L^−1^ [Fe(CN)_6_] ^4−^ (in 1 mol L^−1^ KCl). An AC signal with a 10 mV amplitude and a frequency range of 100 kHz to 0.1 Hz was applied. The acquired impedance spectra (Nyquist plots) were analyzed, and charge transfer resistance (RCT) values were determined using the Randles equivalent circuit model.

## Results and Discussion

**Figure 1:**
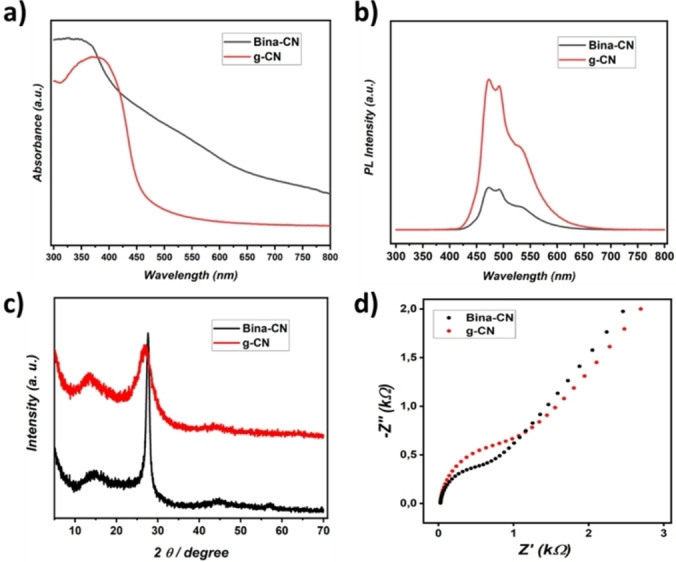
a) Solid state UV‐Vis spectra of Bina‐CN and g‐CN, b) PL spectra of Bina‐CN and g‐CN, c) XRD profiles of Bina‐CN and g‐CN, d) EIS of Bina‐CN and g‐CN.

**Figure 2:**
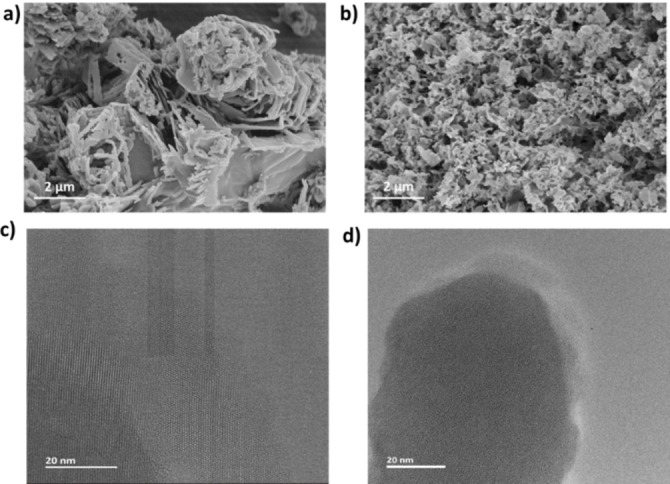
SEM images of a) Bina‐CN and b) g‐CN powders. HRTEM images of c) Bina‐CN and d) g‐CN powders.

Melamine and 1,1′‐Binaphthyl‐2,2′‐diamine (3 : 1) monomer assembly in water can be achieved through hydrogen bonding, and their condensation at 450 °C for 2 hours results in binaphthyl modified carbon nitride (denoted as Bina‐CN, digital image presented in Figure S1), with a solid yield of 44 % compared to 12 % on reference g‐CN sample. An increase in solid yield is attributed to physical behavior of 1,1′‐Binaphthyl‐2,2′‐diamine, which has a melting point of 242 °C and boiling point of 420 °C, hence providing a solvent‐like media followed by dense gas phase formation promoting condensation to a solid state. UV‐Vis spectra confirm a stark difference in light absorption properties of Bina‐CN and g‐CN (Figure 1a) highlighting extended light absorption for the case of Bina‐CN with a corresponding bandgap of 2.19 eV (Figure S2a) compared to 2.78 eV for g‐CN (Figure S2b). Under UV illumination, photoluminescence intensity remains lower for Bina‐CN indicating improved charge separation process compared to g‐CN (Figure 2b).

Crystal plane structure of Bina‐CN is investigated by XRD, which shows typical (100) and (002) planes at 2θ=14.00° and 2θ=27.5°, however much sharper and higher intensity of (002) plane compared to g‐CN is observed, which underlines enhanced interlayer stacking due to binaphthyl edges (Figure 2c). To confirm the influence of comonomer effect, reference experiments using highly aromatic monohydrogen‐containing molecule and polymeric linear amine structures were employed with melamine. Selection of such sophisticated molecules for control experiments is based on their high boiling points that can mimic experimental condition when binaphthyl diamine is used. In case of aromatic system employing 1H‐Benzo[g] indole, carbonization took place (Figure S3) hence this system has not been studied further. This recipe underlines that there should be a balance between benzene rings and amine compounds to attain a carbon nitride structure. On the other hand, experiment employing amine terminated poly(dimethyl siloxane) resulted in white powder with XRD profile very similar to thermally treated melamine^[30]^ (Figure S4), which possibly prevents any condensation reaction due to molecular incompatibility. Additionally, using three times more binaphthyl diamine affords a more carbonaceous material (Figure S5), hence co‐monomer ratio plays a vital role for successful synthesis of carbon nitrides. Lowering binaphthyl diamine (to half) has not shown any notable change compared to reference g‐CN. The electrochemical impedance spectroscopy (EIS) measurements were performed using a redox probe solution to assess the interfacial charge transfer characteristics of the Bina‐CN and g‐CN, placed as modifiers on a glassy carbon electrode surface. The Nyquist plots presented in Figure 1d clearly show that g‐CN exhibits a larger semicircle, indicating a higher charge transfer resistance (RCT), with an RCT value of 888 Ω obtained from the fit. In contrast, Bina‐CN enhances conductivity and facilitates interfacial charge transfer, as evidenced by a smaller semicircle in the plot and lower RCT value of 573 Ω.

Combustive elemental analysis revealed a stark increase in carbon content (50.9 %) of Bina‐CN compared to g‐CN (35.7 %) confirming the enhanced carbon content due to nitrogen‐doped carbon edge formation. However, no detectable surface area was measured through nitrogen sorption experiments, which showed only 2 m^2^ g^−1^ surface area of Bina‐CN (Table S1). Structural difference is notable via FT‐IR analysis which shows a strong signal located at 1600 cm^−1^ in Bina‐CN affiliated to aromatic C=C arising from binaphthyl groups. Notably, the rest of the FTIR spectrum appears coherent with the one acquired for g‐CN, affirming the existence of carbon nitride core structure (Figure S6). TGA is a useful method to evaluate thermal stability of carbon nitride units. Figure S7 depicts TGA of binaphthyl diamine and melamine monomers, showing slightly higher (20 °C) thermal stability for binaphthyl diamine prior to total mass loss at 350 °C. When monomer complex is subjected to TGA (Figure S8), two‐step degradation profile is observed when slow heating rate is applied, which can be attributed to successful complexation. While g‐CN has thermal stability leaving no mass after 700 °C (Figure S9), Bina‐CN possesses high thermal stability up to 510 °C with no mass loss and char yield of 18 % at 1000 °C affording carbonaceous material as evidenced by thermogravimetric analysis (Figure S10). Electron paramagnetic spectrum exhibits a single Lorentzian line between 3430–3450G, at lower magnetic field than reported for g‐CN sample underlining altered electronic state due to nitrogen‐doped carbon edges (Figure S11). Bina‐CN features a significant morphology difference by means of increased crystallinity and order as evidenced by scanning electron microscopy (SEM) and high‐resolution transmission electron microscopy (HRTEM). Nacre‐like stacked crystal sheets are observed as a morphological characteristic of Bina‐CN, whereas no special morphology is observed for g‐CN (Figure 2a–b, Figure S12–13). HRTEM investigations collected from multiple specimen and regions clearly demonstrate a highly ordered nanosheet structure of Bina‐CN (Figure 2c, Figure S14–17) contrary to reference sample of g‐CN (Figure 2d, Figure S18–20). On STEM imaging, high diffraction can be seen on Bina‐CN edges, which hint towards enhanced aromatic density (Figure S21). FFT calculation from selected diffraction area shows 0.34 nm interlayer distance, slightly larger than 0.32 nm conventionally reported for typical carbon nitrides (Figure S22–23).^[31]^ Additionally, in Figure S24 in‐plane diffraction of 0.66 nm was observed and attributed to the in plane diffraction of carbon nitride materials.

Introduction of nitrogen‐doped carbon edges is expected to influence the wettability of carbon nitride structure. Wettability of so‐formed structures was examined via water contact angle measurements performed on carbon nitride deposited on glass surfaces. g‐CN surface has hydrophilic nature with 46.7° water contact angle, whereas Bina‐CN surface exhibits enhanced hydrophobicity with 65.8° water contact angle (Figure S25). We attribute this to the more hydrophobic edges arising upon the copolymerization of melamine and binaphthyl diamine.

**Figure 3:**
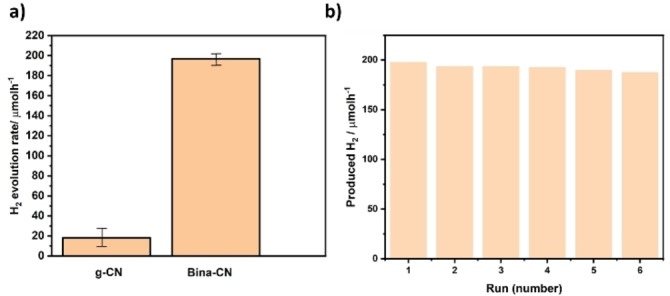
a) Comparison on photocatalytic hydrogen evolution rates of g‐CN and Bina‐CN, b) recyclability of Bina‐CN on 5 consecutive photocatalytic hydrogen evolution runs (duration of each run: 3 hours).

Photocatalytic water splitting performance of Bina‐CN under visible light was conducted in comparison to reference g‐CN with 0.5 wt.% Pt loading (Figure S26) in presence of triethanolamine as hole scavenger. Hydrogen evolution performance of Bina‐CN is significantly higher (almost 9 times) than that of the reference sample, and 194 μmolh^−1^ is comparable to values reported in literature for modified g‐CNs synthesized at 550 °C,^[32]^ despite direct comparison based on produced hydrogen is not a fair comparison as produced hydrogen relies on many factors including reactor setup, temperature, light source and particle dispersion.^[33]^ In our case, enhanced production can be ascribed to extended light absorption covering higher wavelength domain, increased crystallinity as well as charge separation arising from the in‐plane formation of carbon domains with carbon nitride, hence all influencing improved photocatalytic activity. Especially, the influence of carbon nitride crystallinity on hydrogen evolution performance has been well documented in literature, and crystalline ionic carbon nitrides made via salt templating method (Na‐PHI, K‐PHI) outperform traditional carbon nitrides in hydrogen evolution reactions hence underlying the importance of crystallinity in charge transfer processes.^[34]^ Bina‐CN with 0.5 wt.% Pt loading shows high reusability, so that after 6 runs less than 10 % decrease is noted in hydrogen evolution activity (Figure 3b).

In order to gain insight into charge transfer dynamics, time resolved photoluminescence measurements were performed, which shows a significantly reduced exciton lifetime (0.4 vs 1.3 ns) for Bina‐CN (Figure S27a). AQY of Bina‐CN is 9.3 % outperforming g‐CN which has 2.1 % underlining the efficient use of harvested light. This also underlines the influence of enhanced crystallinity and in‐plane heterostructure for improved charge transfer leading to enhanced photocatalytic reaction efficiency for hydrogen evolution. Finally, stability of Bina‐CN photocatalyst is addressed through 5 consecutive hydrogen evolution runs, where each run was 3 hours (Figure S27b). Generated hydrogen is released and reaction continued without adding/separating any constituents. Within the first four runs, a slight decrease in total produced hydrogen amount is observed, which decreases more pronounced in 5^th^ run (around 15 % decrease) indicating moderate level photostability. Whilst such a decrease is not desirable for any photocatalyst, decrease in this case can be attributed to hole scavenger consumption. It is important to note that attempts to load higher Pt leads to leaching, hence for such small surface area sample 0.5 % was found to be optimum. In future, we expect that this strategy can be extended and provide a generation of high surface area Bina‐CN through templating, which can potentially result in improved yields.

## Conclusions

Polymeric carbon nitrides emerge as sustainable metal‐free heterogeneous photocatalysts with visible light activity. The inherent capability for structural modification endows these materials with a spectrum of functionalities, with their role in photocatalytic water splitting featured as a critical area of focus. Despite the inherent advantages, the challenges associated with high‐temperature synthesis and the resulting low solid yields have been affiliated as synthetic problems to tackle. This research demonstrates that a careful tailoring of the monomer composition, particularly through the introduction of binaphthyl diamine as a nanostructure organizer, can substantially lower synthesis temperatures and improve solid yields without sacrificing photocatalytic performance. The integration of binaphthyl diamine enriches the carbon nitride structure by forming nitrogen doped carbon edges, enhancing crystallinity and light absorption properties (with a bandgap of 2.19 eV observed at 450 °C), and achieving hydrogen evolution rates (194 μmol h^−1^) that rival those documented in literature. Monomer composition and their behaviour (such as melting point, boiling point) have major influence on carbon nitride structure and properties. This study further establishes the critical role of monomer selection and their physical properties in determining the final structure and efficacy of carbon nitride photocatalysts. By leveraging melamine as a core source and employing functional molecules with amine functionality as edge modifiers, we unveil a pathway to refine and optimize carbon nitride photocatalysts.

## Supporting Information

Supporting Information is available online at https://doi.org/10.1002/cssc.202400618.

## Conflict of Interests

The authors declare no conflict of interest.

1

## Supporting information

As a service to our authors and readers, this journal provides supporting information supplied by the authors. Such materials are peer reviewed and may be re‐organized for online delivery, but are not copy‐edited or typeset. Technical support issues arising from supporting information (other than missing files) should be addressed to the authors.

Supporting Information

## Data Availability

The data that support the findings of this study are available from the corresponding author upon reasonable request.
