# Supplementary material for: Binaphthyl Mediated Low Temperature Synthesis of Carbon Nitride Photocatalyst for Photocatalytic Hydrogen Evolution
Source: ChemSusChem. 2024 Jul 31;17(22):e202400618. doi: 10.1002/cssc.202400618 (PMC11587680; doi:10.1002/cssc.202400618)
Supplement: Supplementary file 1 — Supporting Information [file CSSC-17-e202400618-s001.pdf]

ChemSusChem

Supporting Information

Binaphthyl Mediated Low Temperature Synthesis of Carbon Nitride Photocatalyst for Photocatalytic Hydrogen Evolution

Simona Baluchová, Sonia Zoltowska, Paolo Giusto,* and Baris Kumru*

Supporting Information For

Binaphthyl Mediated Low Temperature Synthesis of Carbon Nitride Photocatalyst For Photocatalytic Hydrogen Evolution

Simona Baluchová[a], Sonia Zoltowska[b], Paolo Giusto,[b*] Baris Kumru[c*]

a: Department of Analytical Chemistry, Faculty of Science, Charles University , Albertov 6, CZ 128 00, Prague 2, Czech Republic

b: Department of Colloid Chemistry, Max Planck Institute of Colloids and Interfaces, 14476 Potsdam, Germany

c: Aerospace Structures & Materials Department, Faculty of Aerospace Engineering, Delft University of Technology, 2629 HS Delft, The Netherlands

E-mail: paolo.giusto@mpikg.mpg.de b.kumru@tudelft.nl

Contains 17 pages, 27 figures and 1 Table

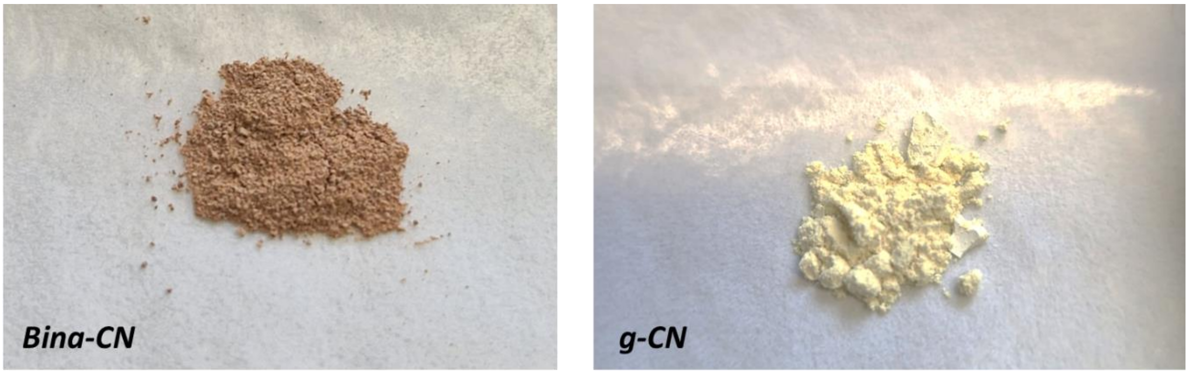

Figure S1. Digital images of Bina-CN and g-CN powder.

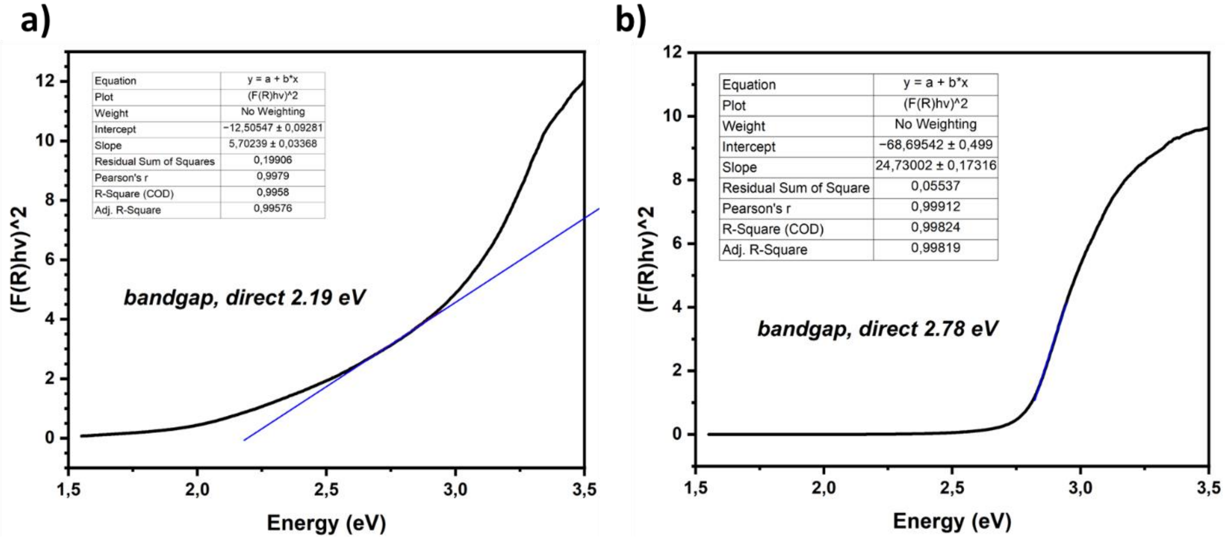

Figure S2. The Tauc plots calculated for a) Bina-CN and b) g-CN.

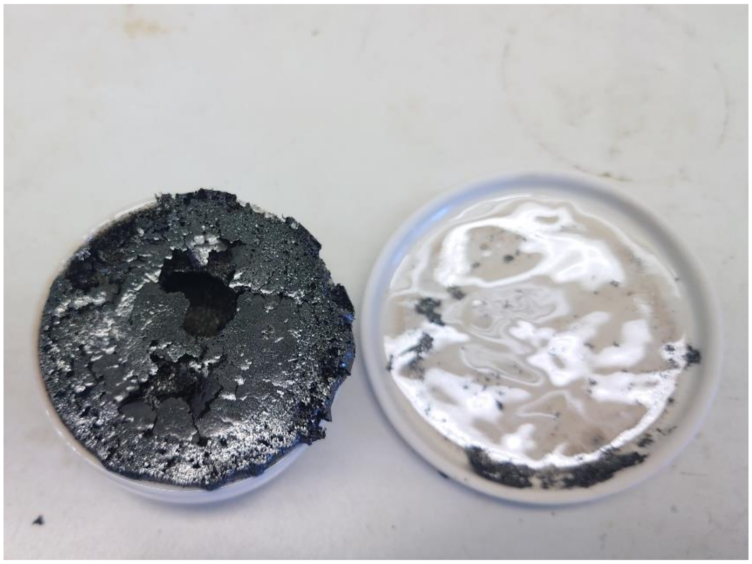

Figure S3. Digital image of BI-CN reference sample affording carbonized structure at 450°C.

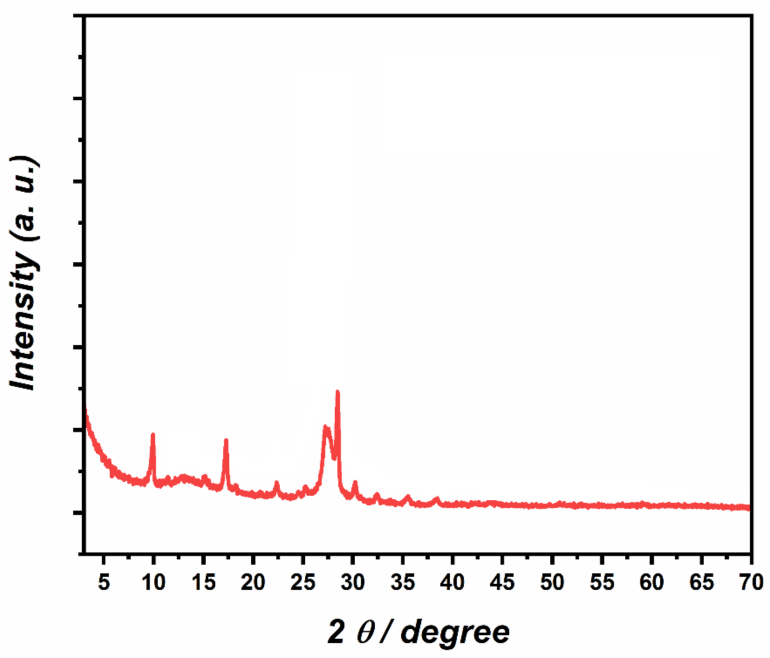

Figure S4. XRD profile of PDMS-CN obtained at 450°C.

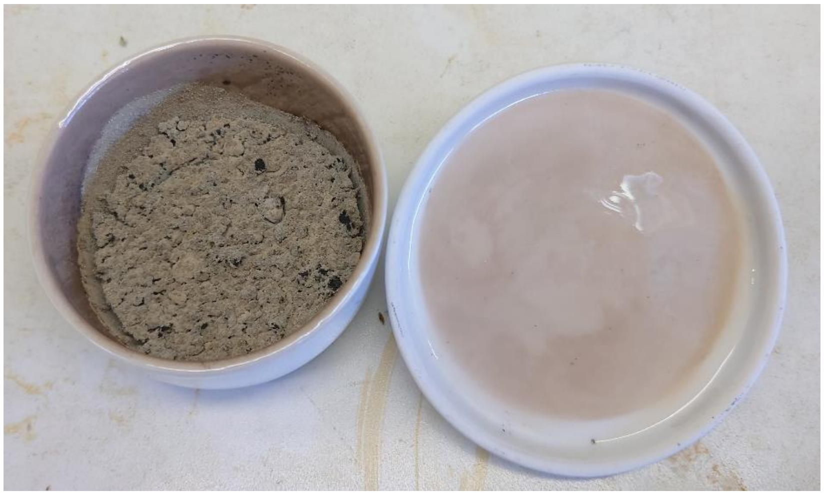

Figure S5. Digital image of 3Bina-CN reference sample affording carbonized structure at 450°C.

Table S1. Elemental composition and surface area of Bina-CN and g-CN. [a]obtained via combustive elemental analysis, [b]obtained via the nitrogen desorption method using BET to evaluate data.

Sample	C (%)[a]	N (%)[a]	H (%)[a]	Surface Area (m^2 g^{-1})[b]
**Bina-CN**	50.9	45.5	3.2	2
**g-CN**	35.7	61	3.1	22

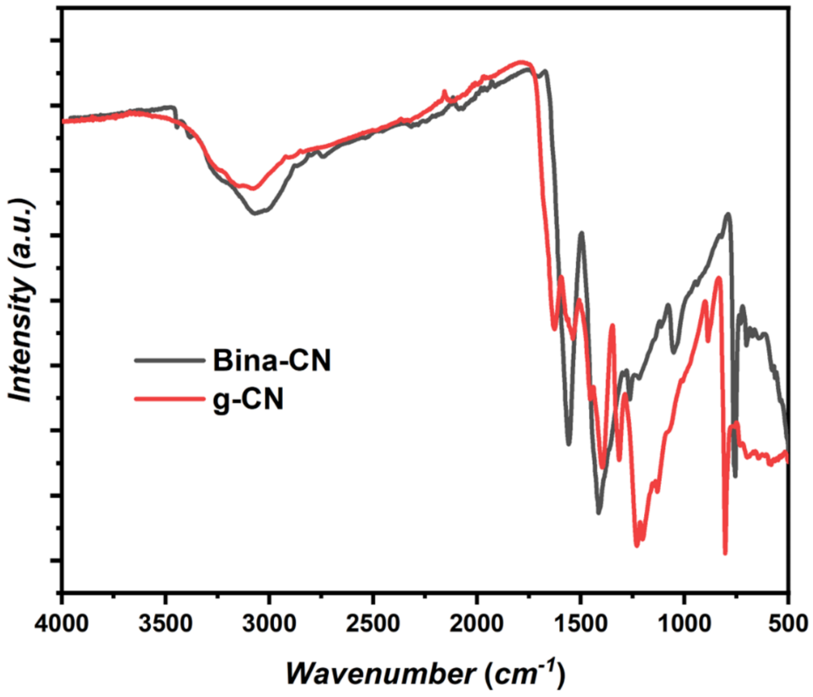

Figure S6. FT-IR spectra of Bina-CN and g-CN.

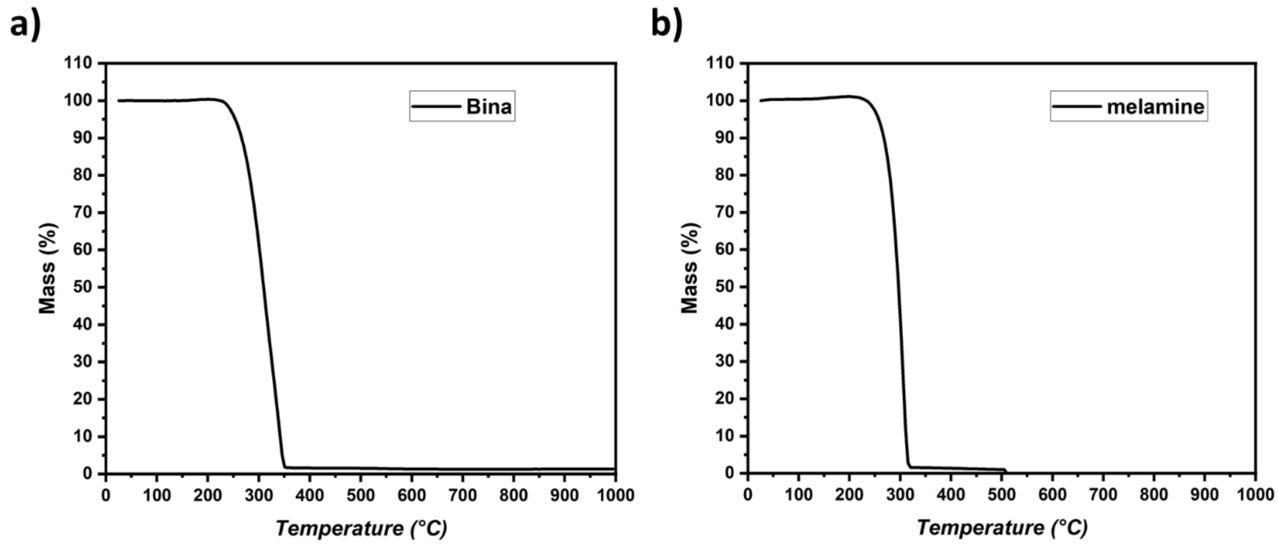

Figure S7. TGA diagrams of a) binaphthyl diamine and b) melamine monomers.

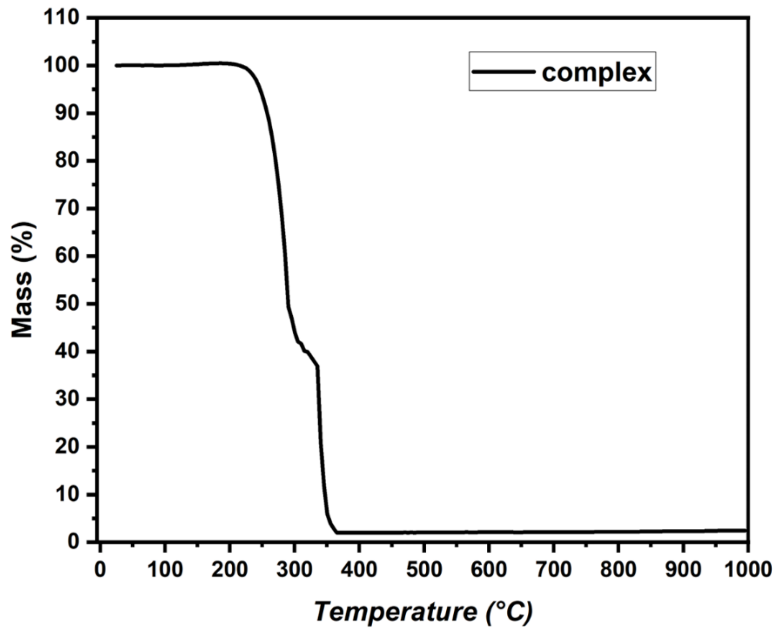

Figure S8. TGA diagram of binaphthyl diamine:melamine complex.

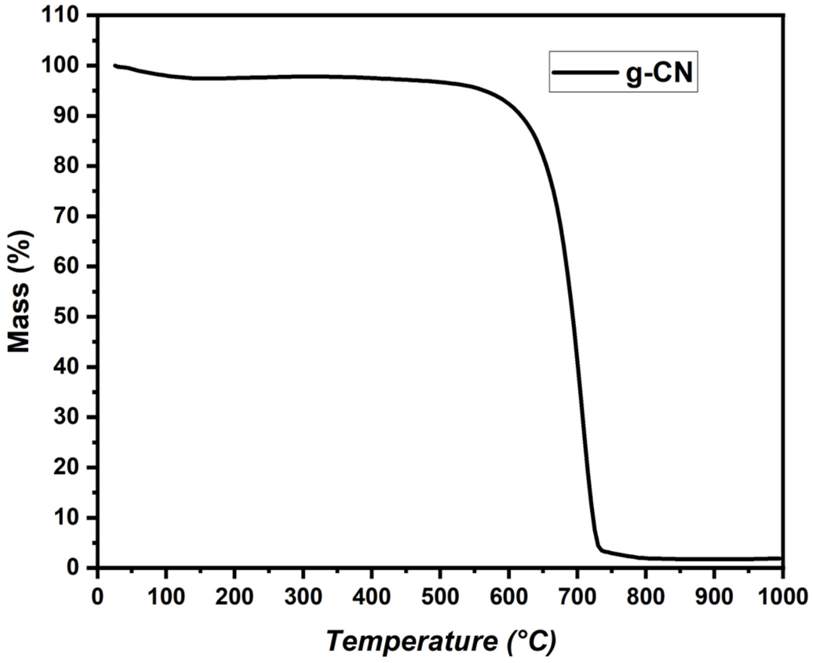

Figure S9. TGA diagram of g-CN.

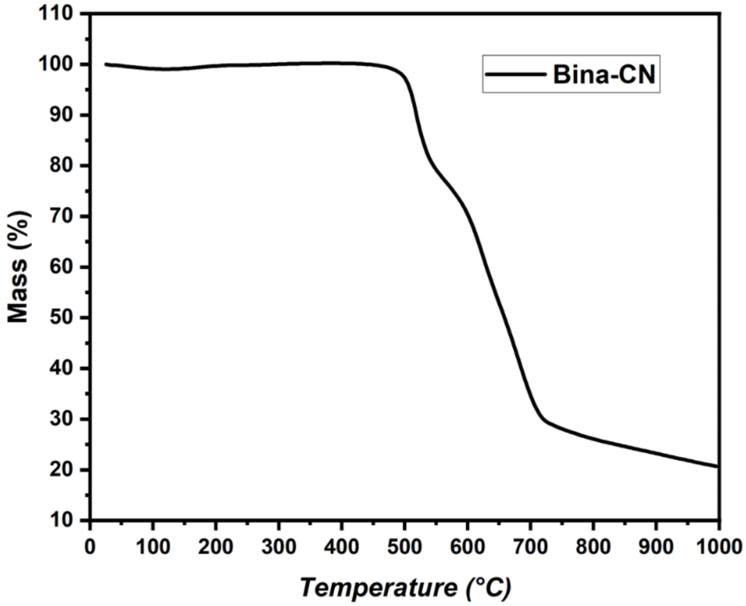

Figure S10. TGA diagram of Bina-CN.

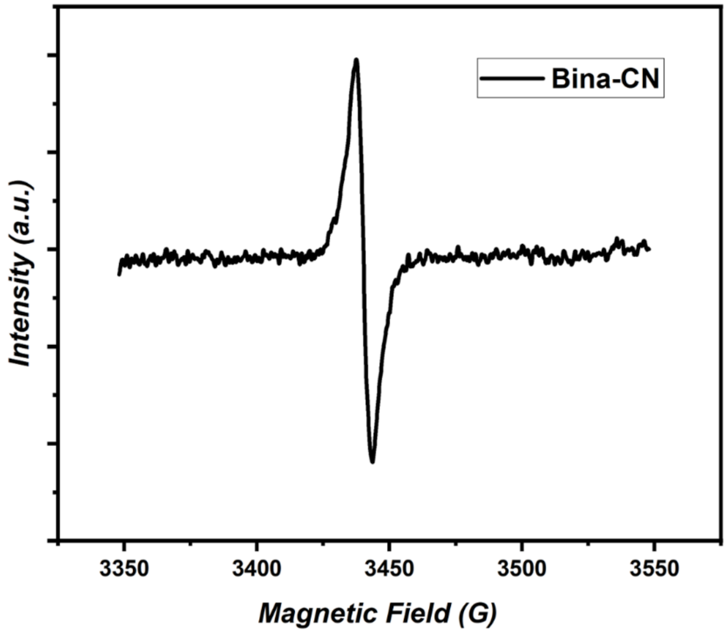

Figure S11. EPR spectrum of Bina-CN.

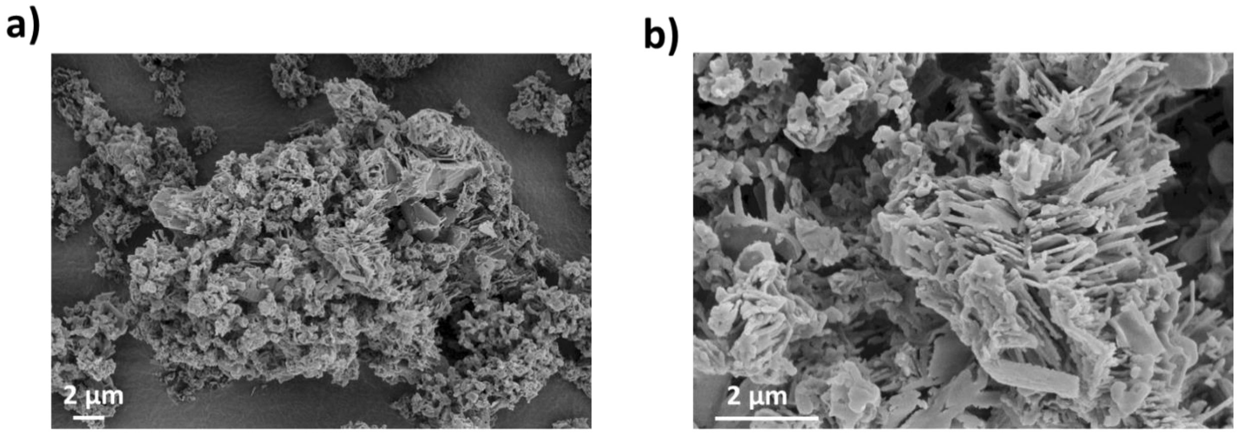

Figure S12. SEM images of Bina-CN powders, a) low magnification and b) high magnification.

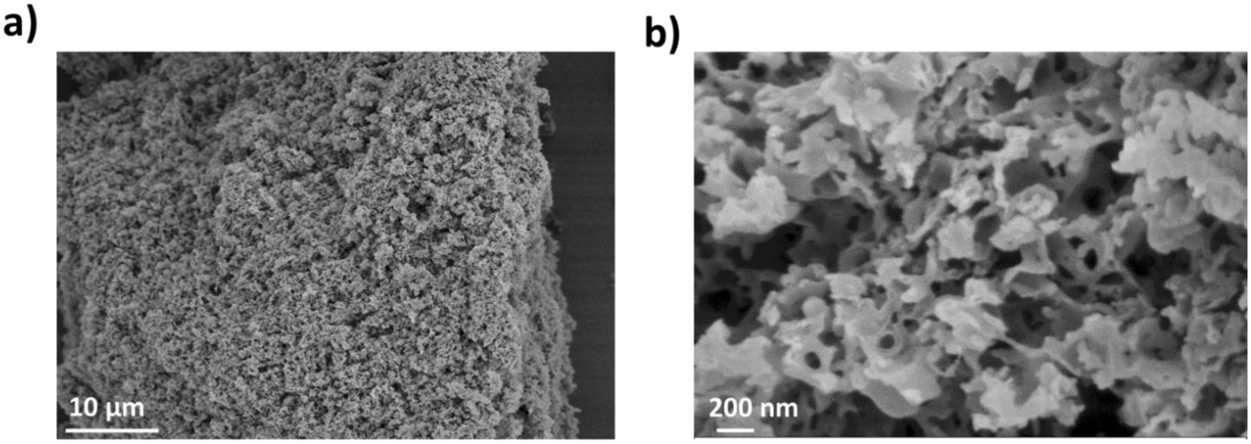

Figure S13. SEM images of g-CN powders, a) low magnification and b) high magnification.

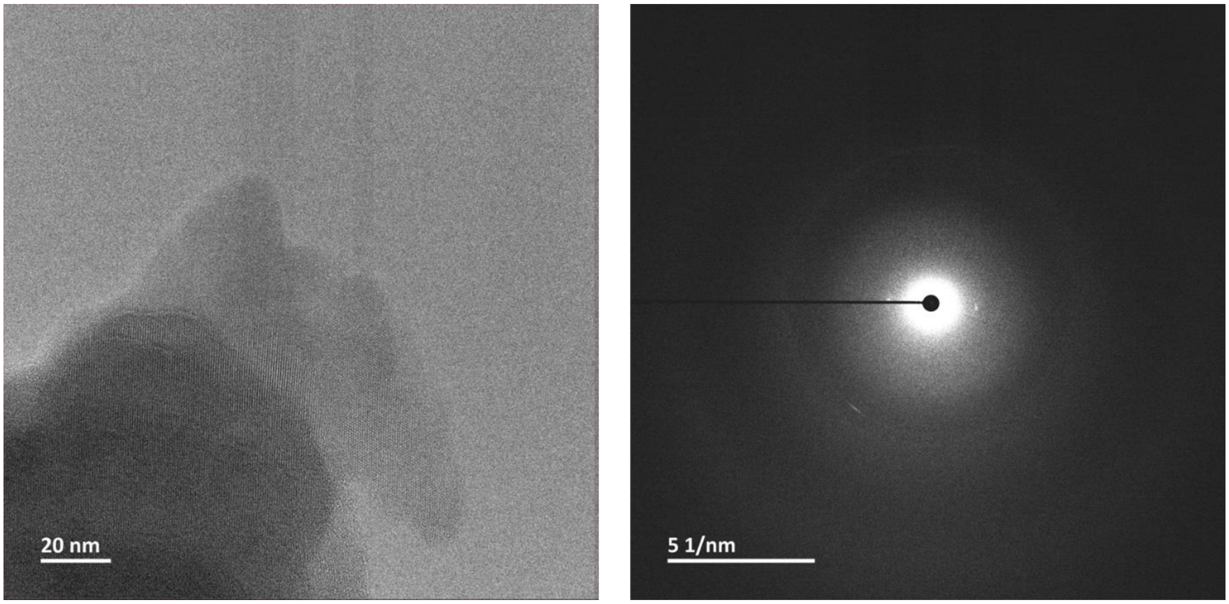

Figure S14. HRTEM image and diffraction spectra of Bina-CN.

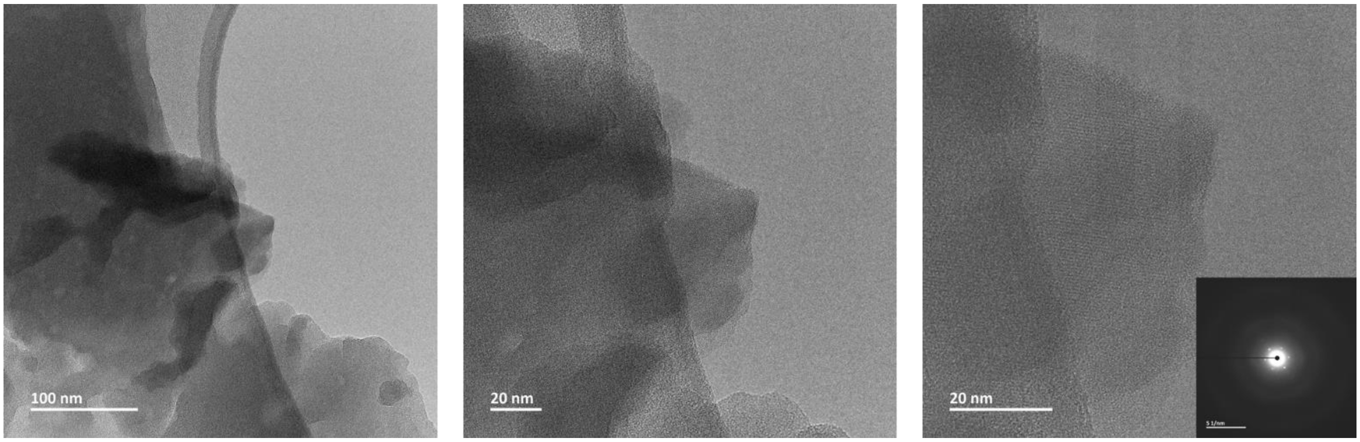

Figure S15. HRTEM images and diffraction spectra of Bina-CN obtained from different location.

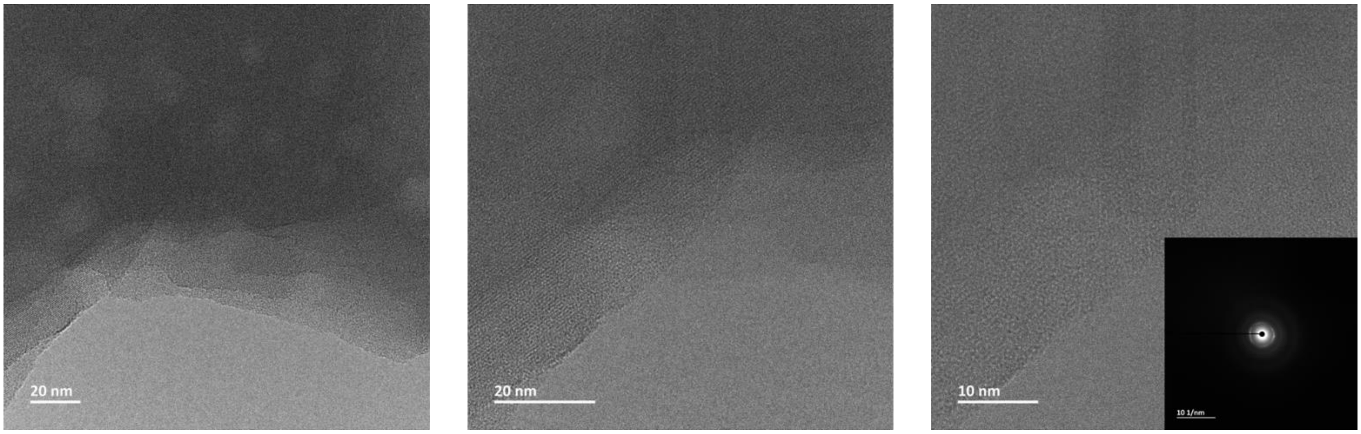

Figure S16. HRTEM images and diffraction spectra of Bina-CN obtained from different location.

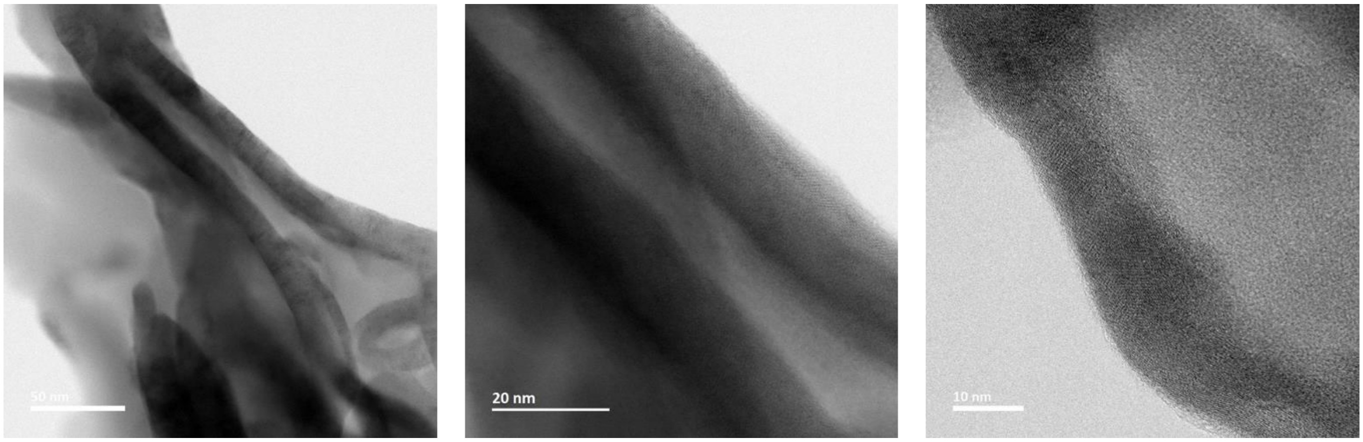

Figure S17. HRTEM images of Bina-CN edges with different magnification values.

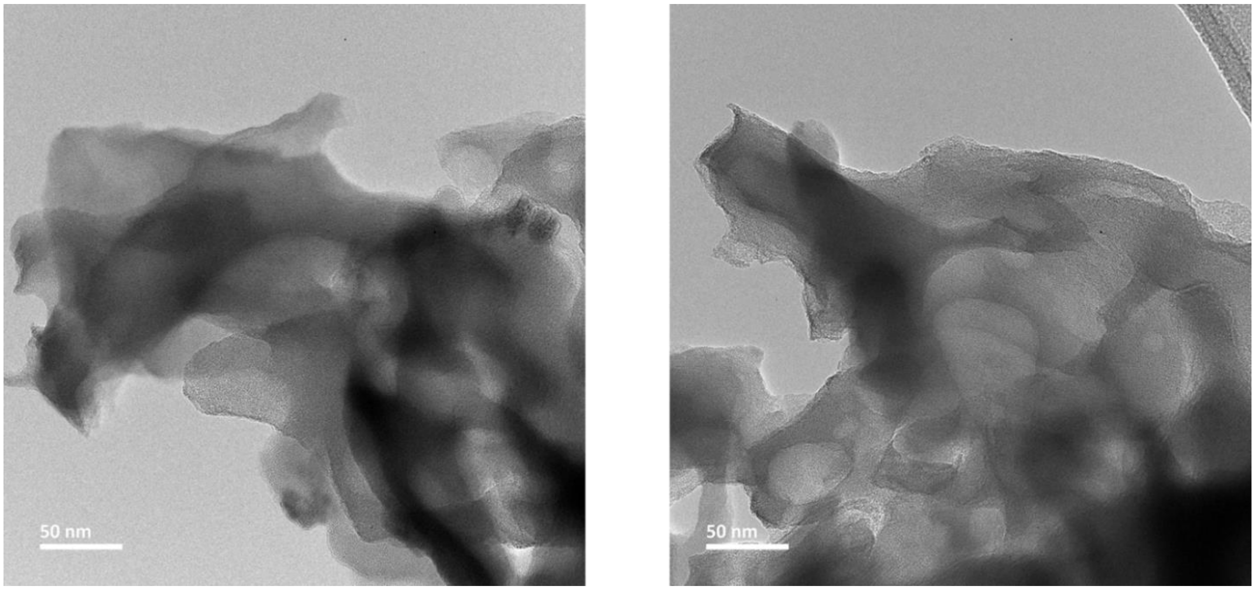

Figure S18. HRTEM images of g-CN powders.

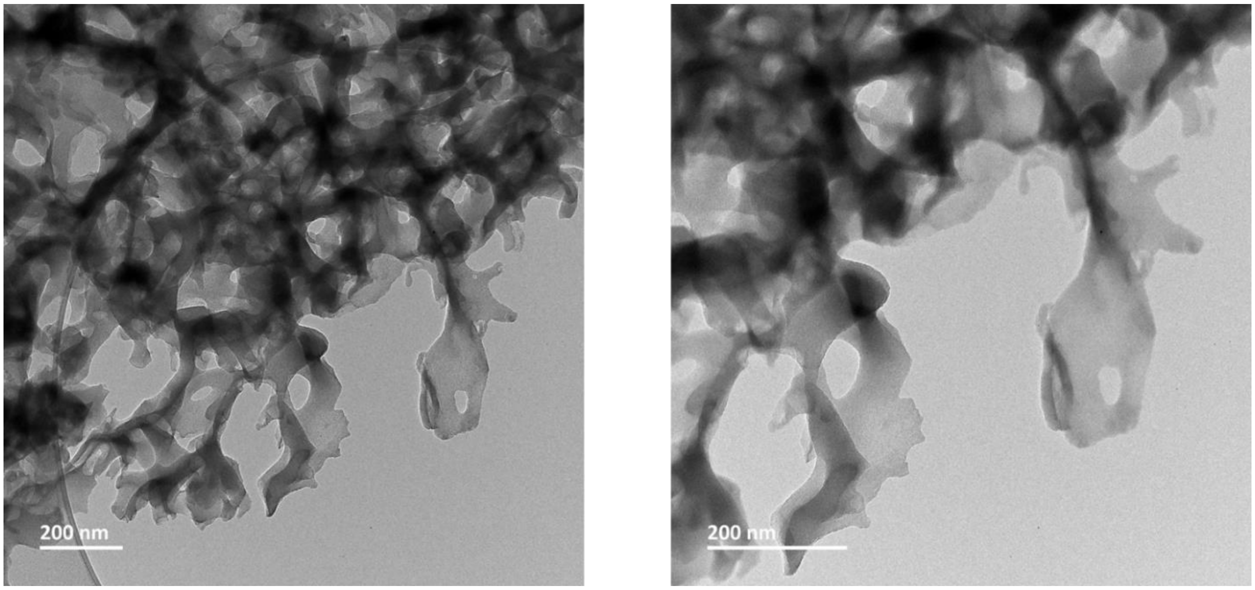

Figure S19. HRTEM images of g-CN obtained from different location.

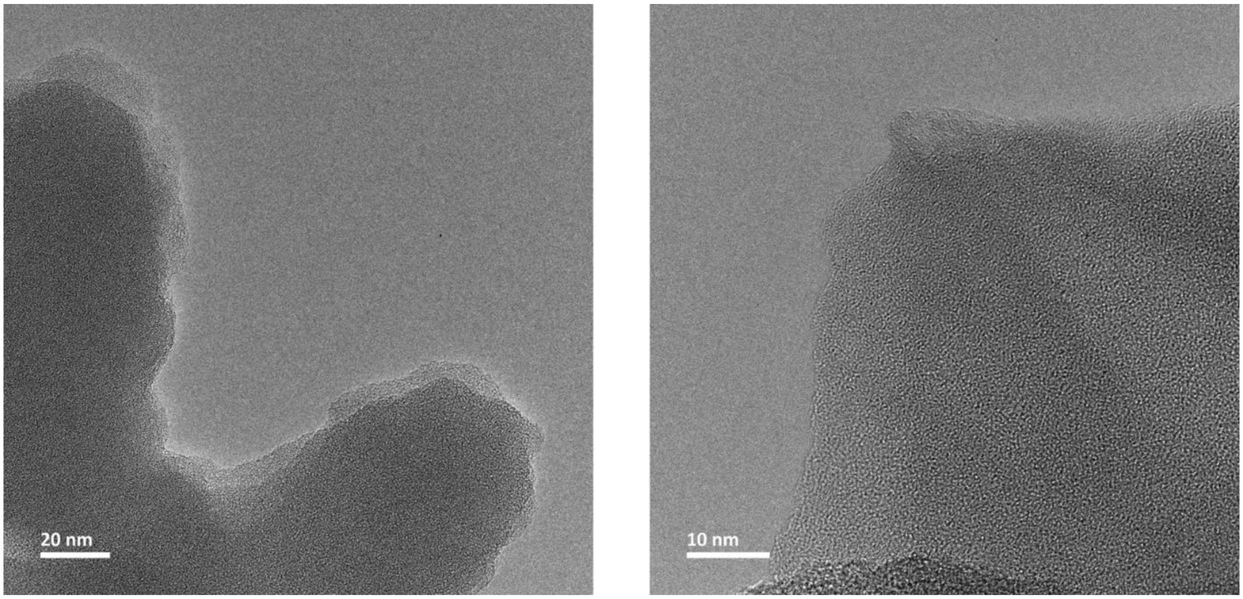

Figure S20. HRTEM images of g-CN obtained from different location focusing on edges.

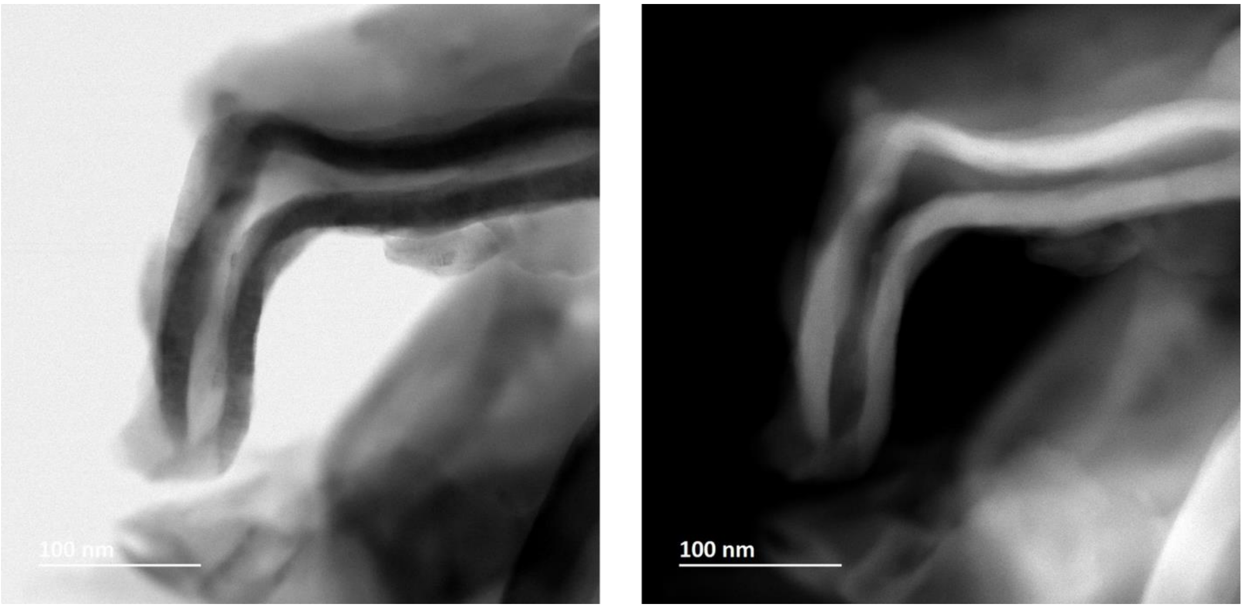

Figure S21. STEM images of Bina-CN edges under brightfield and darkfield.

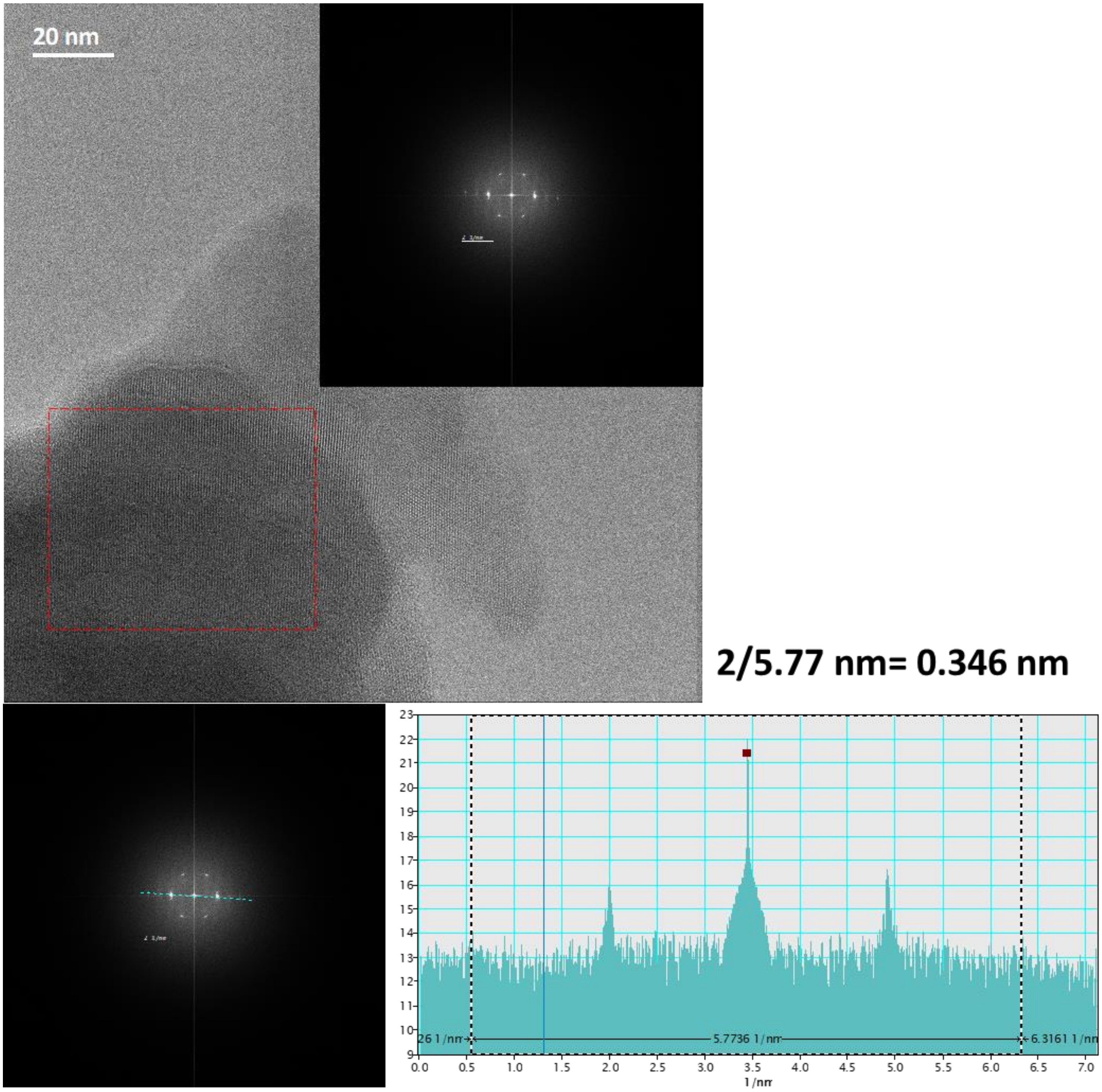

Figure S22. Selected area diffraction and FFT calculation indication 0.346 nm interlayer distance.

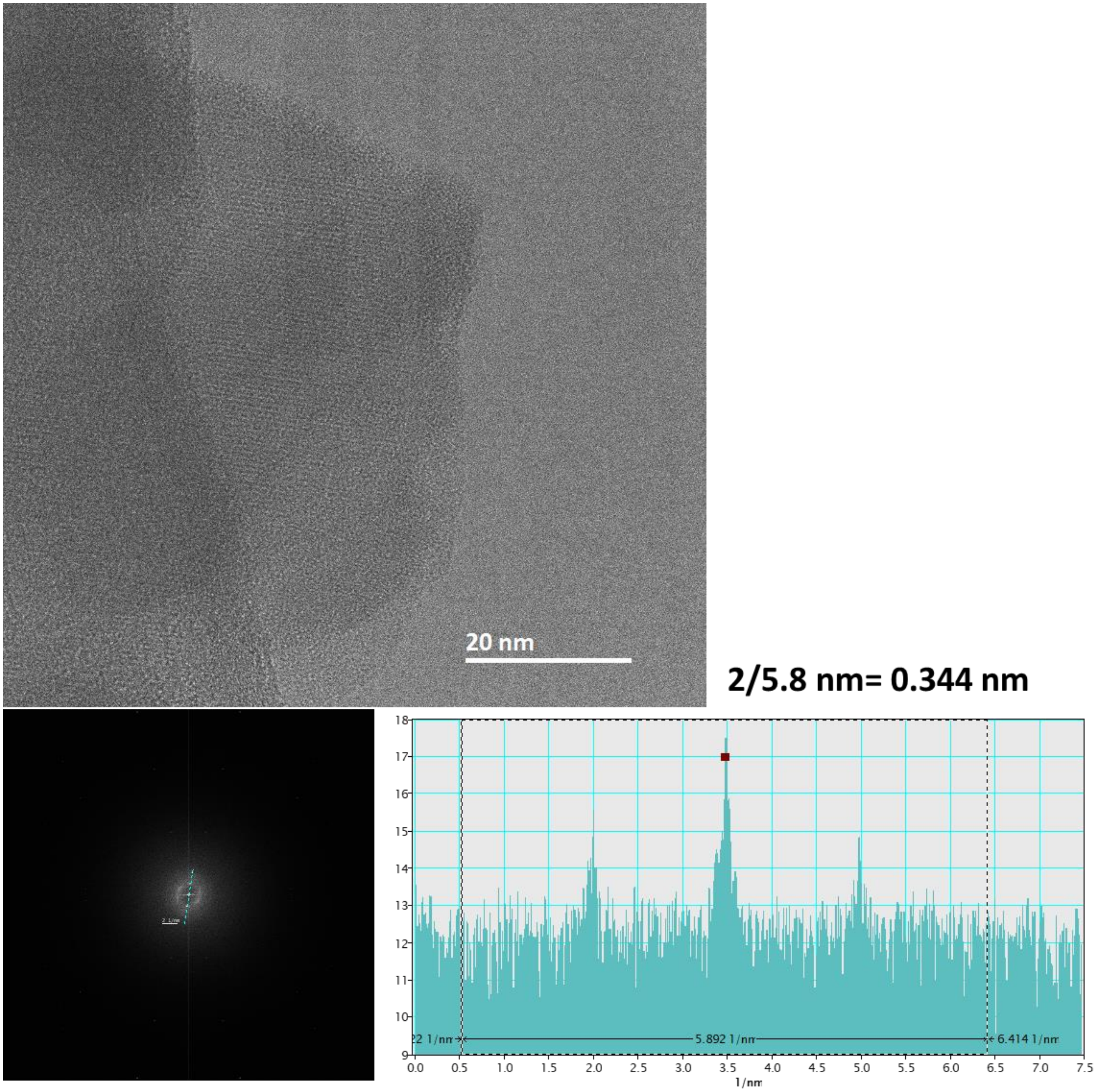

Figure S23. Selected area diffraction and FFT calculation indication 0.344 nm interlayer distance.

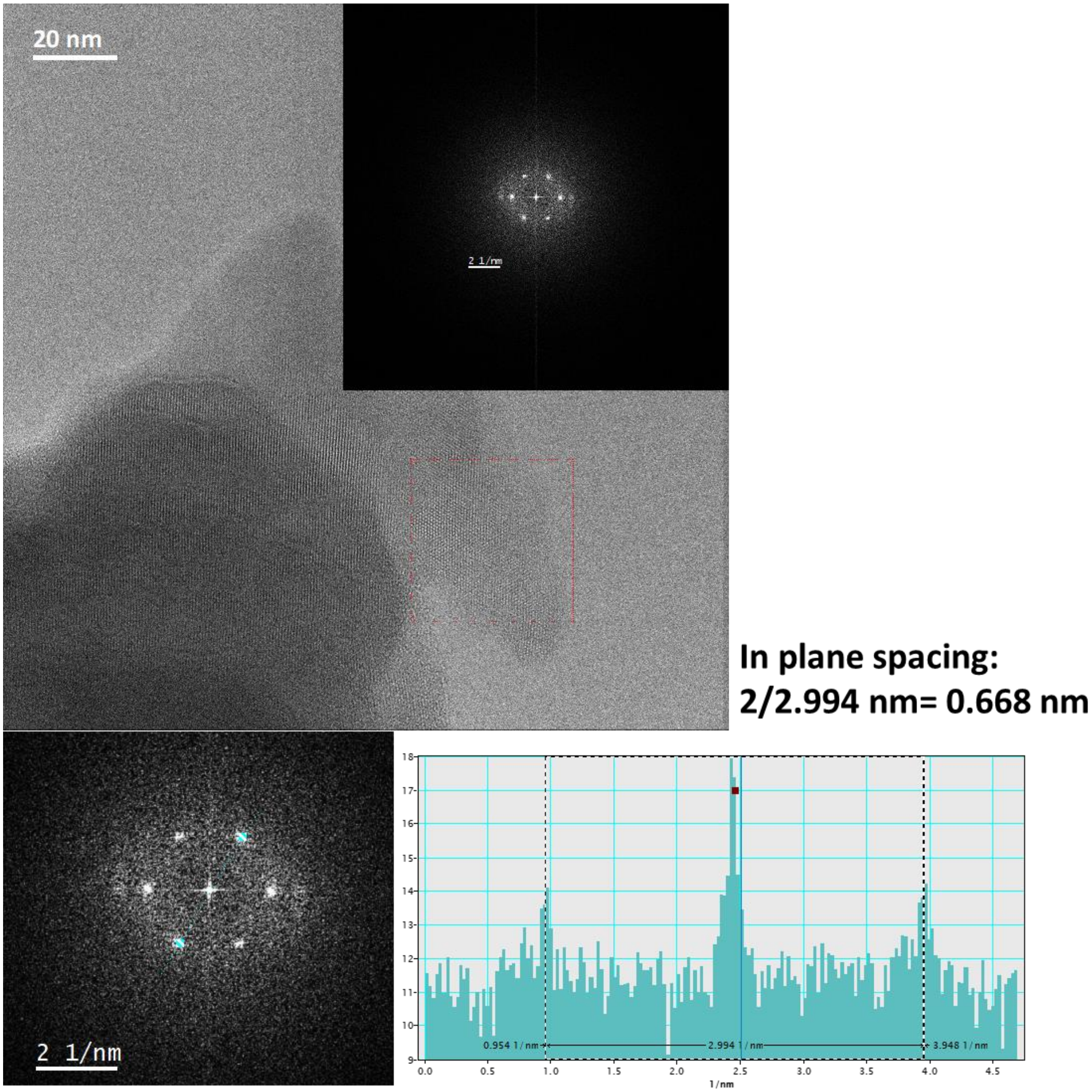

Figure S24. Selected area diffraction and FFT calculation indication 0.66 nm potential in-plane diffraction.

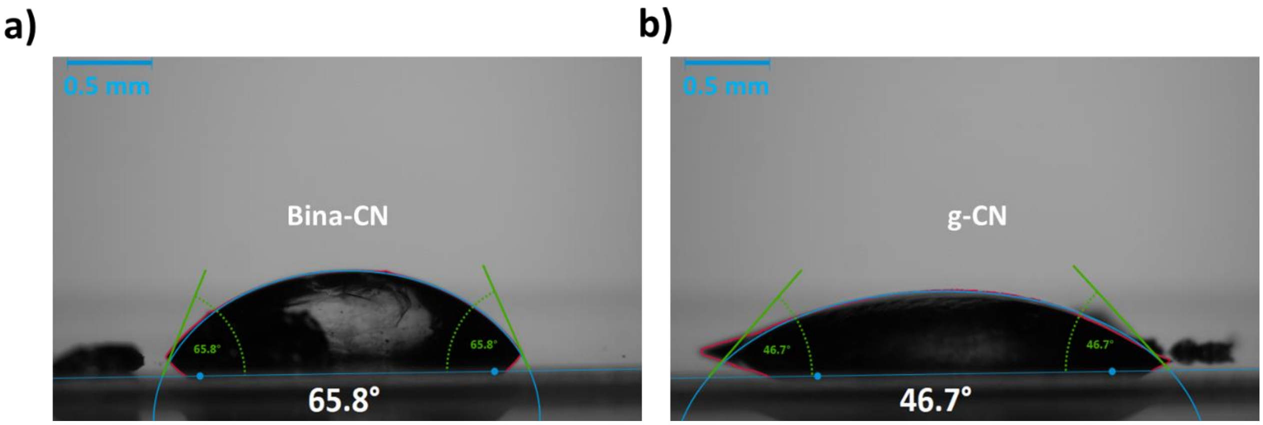

Figure S25. Water contact angle measurements of a) Bina-CN and b) g-CN deposited surfaces.

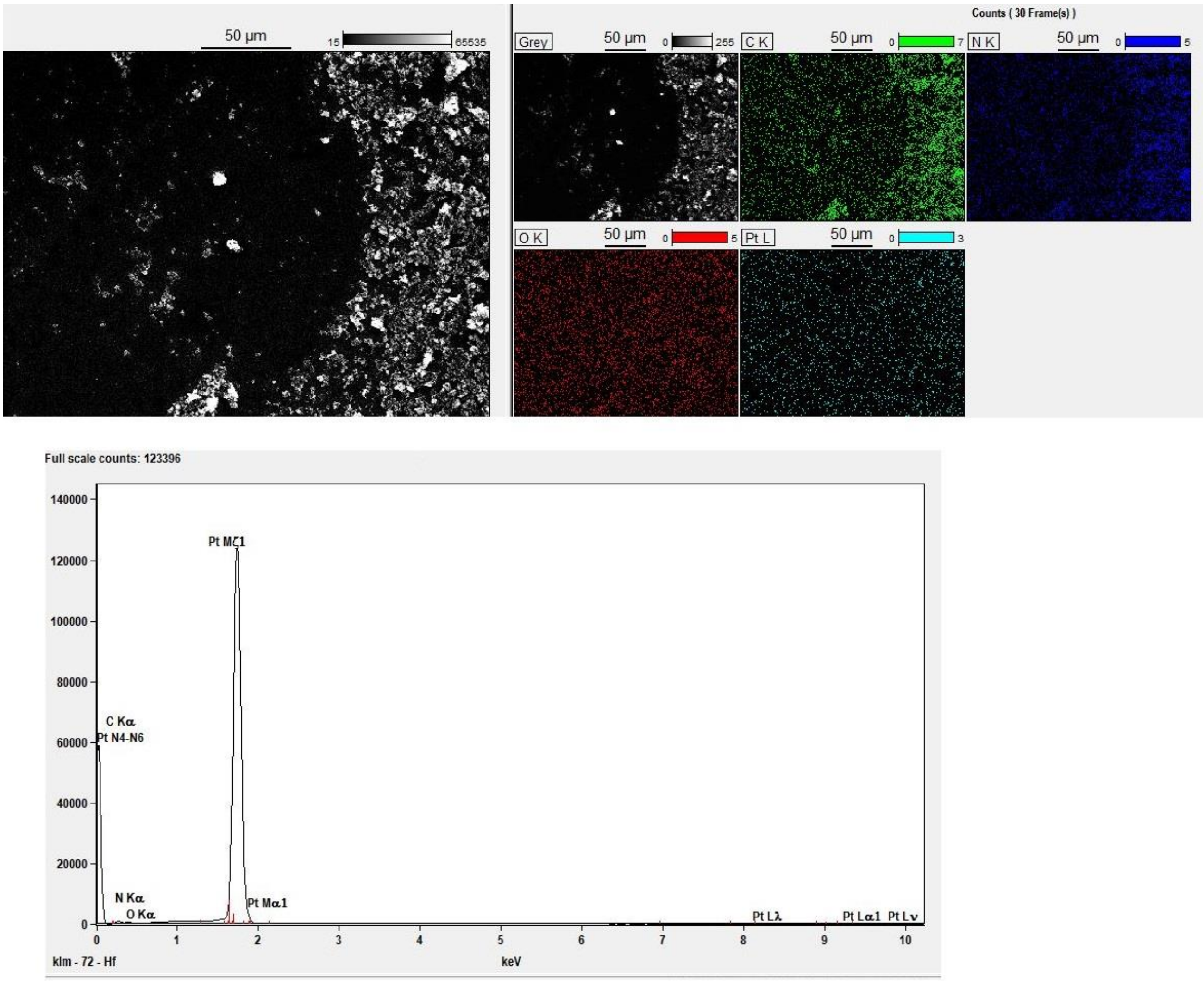

Figure S26. EDX profile of Bina-CN loaded with 0.5 wt.% Pt. (elemental ratios: 54% C, 39% N, 0.6% Pt, 4.1% O)

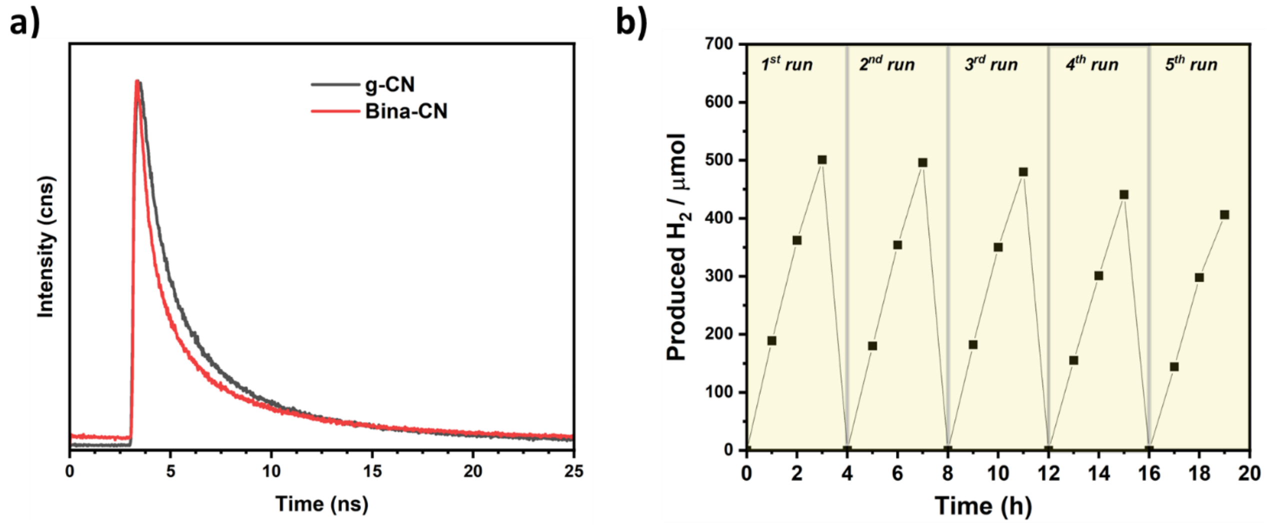

Figure S27. a) Comparative result of time resolved photoluminescence measurement of photocatalysts and b) photostability test of Bina-CN.
